# Editorial: Phylogenetics in the one health context

**DOI:** 10.3389/fmicb.2023.1307616

**Published:** 2023-10-26

**Authors:** Denis Jacob Machado, Larry Jiménez-Ferbans, Kary Ocaña, Adriano de Bernardi Schneider

**Affiliations:** ^1^Department of Bioinformatics and Genomics, College of Computing and Informatics, University of North Carolina at Charlotte, Charlotte, NC, United States; ^2^Universidad del Magdalena, Santa Marta, Colombia; ^3^National Laboratory for Scientific Computing (LNCC), Petrópolis, RJ, Brazil; ^4^Genomics Institute, University of California, Santa Cruz, Santa Cruz, CA, United States

**Keywords:** omics technologies, genomics, one health, phylogenetics, infectious diseases, pathogens, evolution

“Omics” technologies (e.g., genomics, transcriptomics, proteomics, and metabolomics) have revolutionized human health. For example, during the COVID-19 pandemic, genomic epidemiology consolidated as an essential tool to study emerging infectious diseases (EIDs) in humans. However, we can maximize the effectiveness of genomics epidemiology in humans by accounting for diseases' animal and environmental components. The unifying approach to sustainably balance and optimize the health of people, animals, and ecosystems is called “One Health.” Unfortunately, no single “omics” technology is sufficient to understand complex host-pathogen systems in the One Health context. Therefore, this Frontier's Research Topic explores the role of phylogenetics in providing an evolutionary context to integrate “omics” technologies to understand complex biological systems and provide a timely literature foundation for future efforts to bridge the gap between basic research in biodiversity and applied biomedical research on EIDs.

The seven papers in this Research Topic represent a collaborative research effort involving a diverse team of 44 authors from various institutions. These institutions encompass esteemed organizations across Brazil, United States, China, Tunisia, France, Thailand, and the United Kingdom. The extensive representation from various countries underscores the global significance and applicability of the study's findings. This international collaboration harnesses a wealth of expertise and resources to address the research questions at hand, highlighting the broad interest and importance of the subject matter in the worldwide scientific community.

Three papers focused on bacteria of economic or clinical concern. First, Surachat et al. dove into *Weissella cibaria* NH9449's genomics, spotlighting its biotechnological potential and unveiling the strain's self-defense mechanisms and beneficial enzyme-encoding genes. Their evolutionary insight holds promise for applications in the food industry and beyond. Second, Abdallah et al. explored the genomic plasticity of *Coxiella burnetii*, the agent of Q fever. This study uncovers open pangenomes and pathogenicity islands, revealing their adaptability and potential implications for outbreak management and antibiotic resistance. Third, Dekhil and Mardassi unraveled genomic changes driving the success of *Mycobacterium tuberculosis*' Latin American and Mediterranean clonal complex. The authors identified mutations in ESX/Type VII secretion system genes within an evolutionary framework that sheds light on lineage-specific adaptations.

The other four publications investigated viruses. Mao et al. tackled influenza single-site mutation prediction, showing its medical and biotechnological potential. Comparing predictions to subsequent observations highlights the feasibility of estimating mutational profiles in HA antigenic sites. Mao et al. evolutionary perspective aids in preparedness against evolving viral strains. Meanwhile, Perico et al. scrutinized Brazil's SARS-CoV-2 pandemic, highlighting the P.1 (Gamma) variant. The study suggested an external origin and a potential recombinant event, presenting a phylogenetic approach that illuminates the dynamics of pandemic spread. In turn, Sang et al. provided insights into the massive 2019 dengue outbreak in China through phylogenetic analysis, tracing origins and genetic relationships and emphasizing the role of importation. This evolutionary understanding aids in outbreak management and prevention strategies. Finally, Junqueira et al. investigated H1N1pdm09 transmission dynamics from humans to swine in Brazil. These authors observed and discussed evolutionary shifts and antigenic drift patterns, underscoring the dynamic interplay between transmission routes and genetic changes.

The seven articles within this Research Topic contribute significantly to our understanding of how phylogenetics can be integrated into the One Health context, particularly in conjunction with “omics” technologies. Collectively, they address the evolutionary context of complex biological systems and lay a foundation for future research in understanding and combating emerging infectious diseases.

## Author contributions

DJ: Project administration, Writing—original draft, Writing—review & editing. LJ-F: Project administration, Writing—original draft, Writing—review & editing. KO: Project administration, Writing—original draft, Writing—review & editing. AB: Project administration, Writing—original draft, Writing—review & editing.

